# 2,4,5-Tris(pyridin-4-yl)-4,5-dihydro-1,3-oxazole

**DOI:** 10.1107/S1600536812022611

**Published:** 2012-05-26

**Authors:** José J. Campos-Gaxiola, Herbert Höpfl, Gerardo Aguirre, Miguel Parra-Hake

**Affiliations:** a Facultad de Ingeniería Mochis, Universidad Autónoma de Sinaloa, Fuente de Poseidón y Prol. Ángel Flores, 81223 Los Mochis, Sinaloa, Mexico; b Centro de Investigaciones Químicas, Universidad Autónoma del Estado de Morelos, Av. Universidad 1001, 62209 Cuernavaca, Morelos, Mexico; c Centro de Graduados e Investigación del Instituto Tecnológico de Tijuana, Apdo. Postal 1166, 22500 Tijuana, BC, Mexico

## Abstract

In the title compound, C_18_H_14_N_4_O, the mol­ecules are disordered about a crystallographic twofold axis, leading to 50:50 disorder of the O- and N-atom sites within the oxazole ring. As a consequence, symmetry-related oxazole C—N and C—O bonds are averaged. The oxazole ring makes a dihedral angle of 6.920 (1)° with the pyridyl ring in the 2-position and 60.960 (2)° with the pyridyl rings in the 4- and 5-positions.

## Related literature

For background to the synthesis of oxazoles see: Graham (2010 ▶); Aspinall *et al.* (2011 ▶). For the use of pyridyl­oxazole ligands in the construction of metal-organic complexes see: Bettencourt-Dias *et al.* (2010 ▶, 2012 ▶). For the use of tripyridyl ligands in the construction of metal-organic coordination complexes and polymers, see: Campos-Gaxiola *et al.* (2007 ▶, 2008 ▶, 2010 ▶); Liang *et al.* (2008 ▶, 2009 ▶); Yang *et al.* (2010 ▶); Chen *et al.* (2011 ▶).
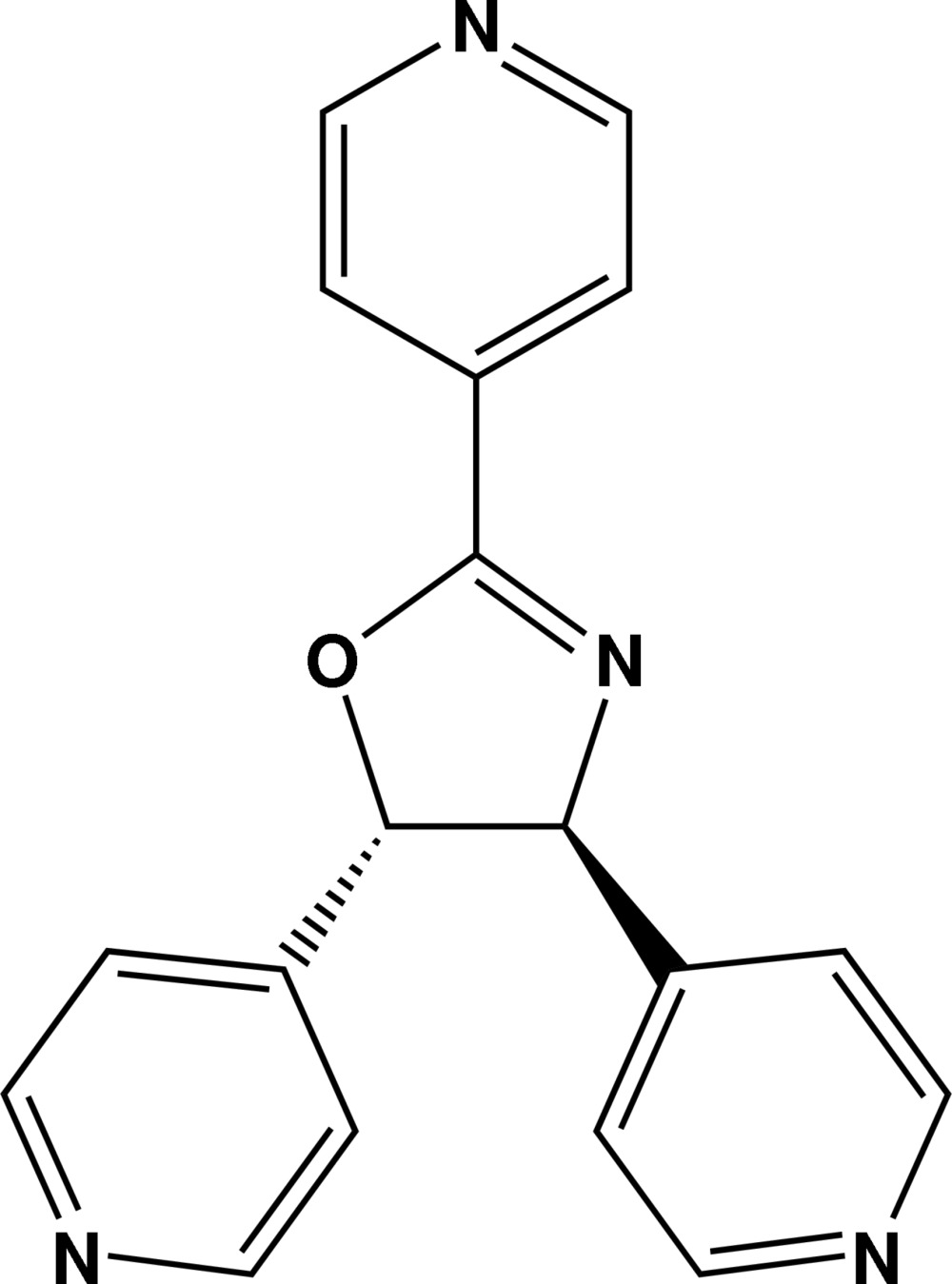


## Experimental

### 

#### Crystal data

C_18_H_14_N_4_O
*M* *_r_* = 302.33
Orthorhombic, 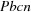

*a* = 15.9777 (13) Å
*b* = 11.4504 (9) Å
*c* = 7.7573 (6) Å
*V* = 1419.21 (19) Å^3^
*Z* = 4
Mo *K*α radiation
μ = 0.09 mm^−1^
*T* = 293 K
0.43 × 0.38 × 0.34 mm

#### Data collection

Bruker SMART CCD area-detector diffractometer
Absorption correction: multi-scan (*SADABS*; Bruker, 2001 ▶) *T*_min_ = 0.962, *T*_max_ = 0.969
12571 measured reflections
1254 independent reflections
1107 reflections with *I* > 2σ(*I*)
*R*_int_ = 0.030

#### Refinement

*R*[*F* ^2^ > 2σ(*F* ^2^)] = 0.039
*wR*(*F* ^2^) = 0.107
*S* = 1.07
1254 reflections
106 parameters
H-atom parameters constrained
Δρ_max_ = 0.16 e Å^−3^
Δρ_min_ = −0.23 e Å^−3^

### 

Data collection: *SMART* (Bruker, 2000 ▶); cell refinement: *SAINT-Plus* (Bruker, 2001 ▶); data reduction: *SAINT-Plus*; program(s) used to solve structure: *SHELXS97* (Sheldrick, 2008 ▶); program(s) used to refine structure: *SHELXL97* (Sheldrick, 2008 ▶); molecular graphics: *SHELXTL* (Sheldrick, 2008 ▶); software used to prepare material for publication: *SHELXTL*.

## Supplementary Material

Crystal structure: contains datablock(s) I, global. DOI: 10.1107/S1600536812022611/pk2402sup1.cif

Structure factors: contains datablock(s) I. DOI: 10.1107/S1600536812022611/pk2402Isup2.hkl

Supplementary material file. DOI: 10.1107/S1600536812022611/pk2402Isup3.cml

Additional supplementary materials:  crystallographic information; 3D view; checkCIF report

## Figures and Tables

**Fig. 1.**
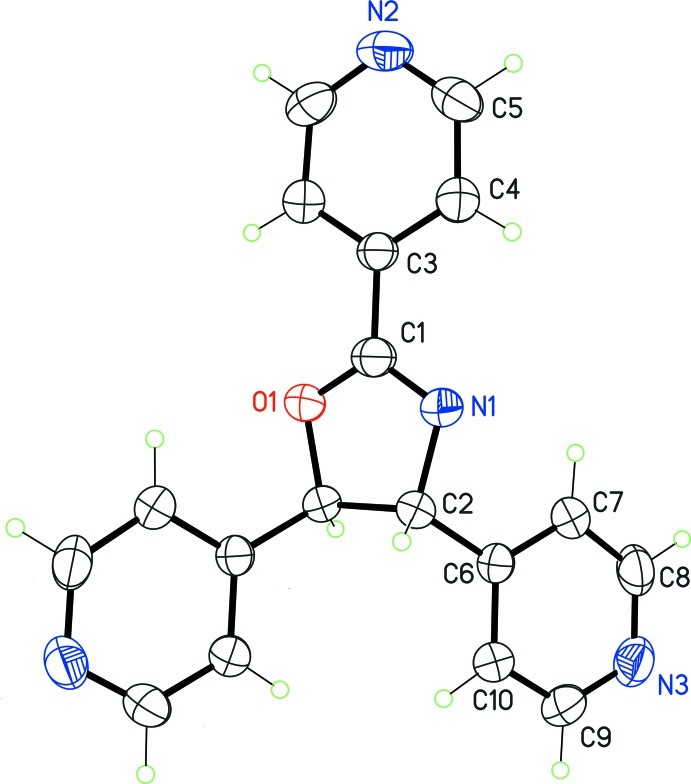
The molecular structure of (I) showing displacement ellipsoids at the 50% probability level. The molecule sits on a crystallographic 2-fold axis (symmetry operation: -x + 1,y,-z + 1/2]), which forces the oxazole ring to be disordered, so that the atoms labelled O1 and N1 occupy sites that are 50% oxygen and 50% nitrogen. These are labelled separately in the diagram to emphasize the chemical nature of the molecules.

## supplementary crystallographic information

### Comment

Several pyridyloxazole derivatives (Bettencourt-Dias *et al.*, 2010 and 2012) have provided effective highly luminescent Ln(III) complexes. Moreover, the coordination chemistry of transition metals with polypyridyl ligands has progressed considerably during the last decade, and has been widely used for the construction of coordination polymers with luminescent properties (Liang *et al.*, 2008, 2009; Chen *et al.*, 2011).

In the course of our studies on transition metal complexes with tripyridyl ligands (Campos-Gaxiola *et al.*, 2008, 2010), we have synthesized the title compound (I) and report its crystal structure here (Fig. 1).

As part of our ongoing research on the design and synthesis of new metal complexes with fluorescent properties, we are interested in using the title compound as a ligand for the synthesis of transition and rare earth metal complexes.

In (I), the molecules are disordered about crystallographic 2-fold-axes, therefore, the C1—N1/C1—O1 and C2—N1/C2—O1 distances are average values. The pyridyl rings attached at positions 4 and 5 show *trans* configuration. The torsion angles for the fragments C(2)^i^—C(2)—C(6)—C(7) and C(2)^i^—C(2)—C(6)—C(10) (symmetry code: (i) -*x* + 1,*y*,-*z* + 1/2) are 104.78 (14)° and -74.55 (15)°, respectively. The oxazole ring forms dihedral angles of 6.920 (2)° with the pyridyl ring in position 2, and 60.960 (1)° with pyridyl rings in positions 4 and 5. No classical hydrogen bonds are observed in the crystal structure.

### Experimental

The synthesis of the title compound included reagent grade starting materials and solvents. A mixture of pyridine-4-carboxaldehyde (5 ml, 0.0531 mol) and ammonium hydroxide (15 ml, 0.3843 mol), dissolved in THF (100 ml) was stirred at 50 °C for 72 h and the solvent removed under reduced pressure. The remaining solid was re-crystallized in methanol, providing colorless crystals. Yield (3.5 g, 60%). IR (KBr): 3154, 3035, 3005, 2889, 1660, 1596, 1557, 1486, 1492, 1412, 1333, 1290, 1077, 992, 823, 677 cm^-1^.

### Refinement

All H atoms on C atoms were positioned geometrically and refined as riding atoms, with (C—H = 0.93 Å) and *U*_iso_(H) = 1.2 *U*_eq_(C). The molecules are disordered over crystallographic 2-fold axes, therefore, the C1—N1/C1—O1 and C2—N1/C2—O1 distances are average values. The EXYZ and EADP constraint instructions in SHELXL-97 were used for atoms N1 and O1 in order to model the disorder properly during the refinement.

### Crystal data

**Table:**

C_18_H_14_N_4_O	*F*(000) = 632
*M**_r_* = 302.33	*D*_x_ = 1.415 Mg m^−^^3^
Orthorhombic, *P**b**c**n*	Mo *K*α radiation, λ = 0.71073 Å
Hall symbol: -P 2n 2ab	Cell parameters from 5620 reflections
*a* = 15.9777 (13) Å	θ = 2.2–28.3°
*b* = 11.4504 (9) Å	µ = 0.09 mm^−^^1^
*c* = 7.7573 (6) Å	*T* = 293 K
*V* = 1419.21 (19) Å^3^	Rectangular prism, colorless
*Z* = 4	0.43 × 0.38 × 0.34 mm

### Data collection

**Table:**

Bruker SMART CCD area-detector diffractometer	1254 independent reflections
Radiation source: fine-focus sealed tube	1107 reflections with *I* > 2σ(*I*)
Graphite monochromator	*R*_int_ = 0.030
φ and ω scans	θ_max_ = 25.0°, θ_min_ = 2.2°
Absorption correction: multi-scan (*SADABS*; Bruker, 2001)	*h* = −19→19
*T*_min_ = 0.962, *T*_max_ = 0.969	*k* = −13→13
12571 measured reflections	*l* = −9→9

### Refinement

**Table:**

Refinement on *F*^2^	Primary atom site location: structure-invariant direct methods
Least-squares matrix: full	Secondary atom site location: difference Fourier map
*R*[*F*^2^ > 2σ(*F*^2^)] = 0.039	Hydrogen site location: inferred from neighbouring sites
*wR*(*F*^2^) = 0.107	H-atom parameters constrained
*S* = 1.07	*w* = 1/[σ^2^(*F*_o_^2^) + (0.0526*P*)^2^ + 0.3873*P*] where *P* = (*F*_o_^2^ + 2*F*_c_^2^)/3
1254 reflections	(Δ/σ)_max_ < 0.001
106 parameters	Δρ_max_ = 0.16 e Å^−^^3^
0 restraints	Δρ_min_ = −0.23 e Å^−^^3^

### Special details

**Table:**

Geometry. All e.s.d.'s (except the e.s.d. in the dihedral angle between two l.s. planes) are estimated using the full covariance matrix. The cell e.s.d.'s are taken into account individually in the estimation of e.s.d.'s in distances, angles and torsion angles; correlations between e.s.d.'s in cell parameters are only used when they are defined by crystal symmetry. An approximate (isotropic) treatment of cell e.s.d.'s is used for estimating e.s.d.'s involving l.s. planes.
Refinement. Refinement of *F*^2^ against ALL reflections. The weighted *R*-factor *wR* and goodness of fit *S* are based on *F*^2^, conventional *R*-factors *R* are based on *F*, with *F* set to zero for negative *F*^2^. The threshold expression of *F*^2^ > σ(*F*^2^) is used only for calculating *R*-factors(gt) *etc*. and is not relevant to the choice of reflections for refinement. *R*-factors based on *F*^2^ are statistically about twice as large as those based on *F*, and *R*- factors based on ALL data will be even larger.

### Fractional atomic coordinates and isotropic or equivalent isotropic displacement parameters (Å^2^)

**Table:**

	*x*	*y*	*z*	*U*_iso_*/*U*_eq_	Occ. (<1)
C1	0.5000	0.86189 (17)	0.2500	0.0382 (5)	
N1	0.44050 (7)	0.80349 (9)	0.17275 (14)	0.0380 (3)	0.50
O1	0.44050 (7)	0.80349 (9)	0.17275 (14)	0.0380 (3)	0.50
C2	0.45914 (8)	0.68010 (11)	0.19468 (17)	0.0327 (3)	
H2	0.4713	0.6458	0.0817	0.039*	
N2	0.5000	1.23405 (16)	0.2500	0.0507 (5)	
N3	0.25339 (8)	0.49131 (13)	0.42490 (18)	0.0509 (4)	
C3	0.5000	0.99035 (17)	0.2500	0.0327 (4)	
C4	0.43313 (9)	1.05212 (13)	0.18356 (19)	0.0405 (4)	
H4	0.3870	1.0136	0.1374	0.049*	
C5	0.43658 (11)	1.17225 (14)	0.1875 (2)	0.0480 (4)	
H5	0.3911	1.2131	0.1431	0.058*	
C6	0.38645 (8)	0.61604 (12)	0.27474 (18)	0.0334 (3)	
C7	0.31909 (9)	0.67265 (13)	0.34906 (19)	0.0406 (4)	
H7	0.3168	0.7538	0.3508	0.049*	
C8	0.25525 (9)	0.60737 (15)	0.4207 (2)	0.0487 (4)	
H8	0.2105	0.6474	0.4694	0.058*	
C9	0.31870 (10)	0.43864 (13)	0.3541 (2)	0.0500 (4)	
H9	0.3197	0.3574	0.3560	0.060*	
C10	0.38510 (9)	0.49502 (13)	0.2781 (2)	0.0423 (4)	
H10	0.4287	0.4524	0.2295	0.051*	

### Atomic displacement parameters (Å^2^)

**Table:**

	*U*^11^	*U*^22^	*U*^33^	*U*^12^	*U*^13^	*U*^23^
C1	0.0407 (12)	0.0313 (10)	0.0426 (11)	0.000	0.0142 (9)	0.000
N1	0.0352 (6)	0.0292 (6)	0.0495 (7)	−0.0013 (4)	−0.0024 (5)	0.0045 (5)
O1	0.0352 (6)	0.0292 (6)	0.0495 (7)	−0.0013 (4)	−0.0024 (5)	0.0045 (5)
C2	0.0306 (7)	0.0276 (7)	0.0399 (7)	0.0015 (6)	0.0005 (6)	−0.0017 (6)
N2	0.0645 (13)	0.0313 (9)	0.0562 (12)	0.000	0.0062 (10)	0.000
N3	0.0354 (7)	0.0536 (8)	0.0637 (9)	−0.0076 (6)	−0.0014 (6)	0.0109 (7)
C3	0.0340 (10)	0.0289 (10)	0.0352 (10)	0.000	0.0077 (8)	0.000
C4	0.0365 (8)	0.0394 (8)	0.0456 (9)	−0.0001 (6)	0.0006 (6)	−0.0014 (7)
C5	0.0526 (9)	0.0391 (8)	0.0523 (9)	0.0118 (7)	0.0019 (7)	0.0053 (7)
C6	0.0289 (7)	0.0326 (7)	0.0387 (7)	−0.0005 (6)	−0.0048 (6)	−0.0003 (6)
C7	0.0322 (8)	0.0360 (8)	0.0536 (9)	0.0030 (6)	−0.0006 (7)	0.0008 (7)
C8	0.0296 (8)	0.0565 (10)	0.0602 (10)	0.0049 (7)	0.0043 (7)	0.0032 (8)
C9	0.0430 (9)	0.0354 (8)	0.0717 (11)	−0.0077 (7)	−0.0053 (8)	0.0049 (7)
C10	0.0342 (7)	0.0329 (7)	0.0598 (9)	−0.0008 (6)	0.0004 (7)	−0.0039 (7)

### Geometric parameters (Å, º)

**Table:**

C1—N1	1.3077 (14)	C3—C4^i^	1.3811 (18)
C1—O1^i^	1.3077 (14)	C4—C5	1.377 (2)
C1—C3	1.471 (3)	C4—H4	0.9300
N1—C2	1.4539 (17)	C5—H5	0.9300
C2—C6	1.5076 (19)	C6—C7	1.382 (2)
C2—C2^i^	1.563 (3)	C6—C10	1.386 (2)
C2—H2	0.9800	C7—C8	1.381 (2)
N2—C5	1.3277 (19)	C7—H7	0.9300
N2—C5^i^	1.3277 (19)	C8—H8	0.9300
N3—C9	1.324 (2)	C9—C10	1.375 (2)
N3—C8	1.330 (2)	C9—H9	0.9300
C3—C4	1.3811 (18)	C10—H10	0.9300
			
N1—C1—O1^i^	118.50 (17)	N2—C5—C4	124.82 (15)
N1—C1—C3	120.75 (9)	N2—C5—H5	117.6
O1^i^—C1—C3	120.75 (9)	C4—C5—H5	117.6
N1^i^—C1—C3	120.75 (9)	C7—C6—C10	116.67 (13)
C1—N1—C2	107.12 (12)	C7—C6—C2	122.92 (12)
N1—C2—C6	111.30 (11)	C10—C6—C2	120.41 (12)
N1—C2—C2^i^	103.62 (7)	C8—C7—C6	119.27 (14)
C6—C2—C2^i^	114.67 (12)	C8—C7—H7	120.4
N1—C2—H2	109.0	C6—C7—H7	120.4
C6—C2—H2	109.0	N3—C8—C7	124.56 (15)
C2^i^—C2—H2	109.0	N3—C8—H8	117.7
C5—N2—C5^i^	115.59 (19)	C7—C8—H8	117.7
C9—N3—C8	115.29 (13)	N3—C9—C10	124.89 (14)
C4—C3—C4^i^	118.38 (19)	N3—C9—H9	117.6
C4—C3—C1	120.81 (9)	C10—C9—H9	117.6
C4^i^—C3—C1	120.81 (9)	C9—C10—C6	119.31 (14)
C5—C4—C3	118.19 (15)	C9—C10—H10	120.3
C5—C4—H4	120.9	C6—C10—H10	120.3
C3—C4—H4	120.9		
			
O1^i^—C1—N1—C2	−0.62 (6)	C3—C4—C5—N2	0.4 (2)
N1^i^—C1—N1—C2	−0.62 (6)	N1—C2—C6—C7	−12.42 (18)
C3—C1—N1—C2	179.38 (6)	C2^i^—C2—C6—C7	104.78 (14)
C1—N1—C2—C6	125.19 (10)	N1—C2—C6—C10	168.25 (12)
C1—N1—C2—C2^i^	1.45 (15)	C2^i^—C2—C6—C10	−74.55 (15)
N1—C1—C3—C4	6.47 (9)	C10—C6—C7—C8	−0.2 (2)
O1^i^—C1—C3—C4	−173.53 (9)	C2—C6—C7—C8	−179.56 (13)
N1^i^—C1—C3—C4	−173.53 (9)	C9—N3—C8—C7	0.1 (2)
N1—C1—C3—C4^i^	−173.53 (9)	C6—C7—C8—N3	0.3 (2)
O1^i^—C1—C3—C4^i^	6.47 (9)	C8—N3—C9—C10	−0.7 (3)
N1^i^—C1—C3—C4^i^	6.47 (9)	N3—C9—C10—C6	0.8 (3)
C4^i^—C3—C4—C5	−0.17 (10)	C7—C6—C10—C9	−0.3 (2)
C1—C3—C4—C5	179.83 (10)	C2—C6—C10—C9	179.05 (14)
C5^i^—N2—C5—C4	−0.19 (11)		

Symmetry code: (i) −*x*+1, *y*, −*z*+1/2.
